# Vietnamese University Students’ Perceptions and Attitudes Toward Participation in Clinical Research: Mixed Methods Study

**DOI:** 10.2196/86269

**Published:** 2026-02-12

**Authors:** Chi Le Phuong, Vy Pham-Tram, Hong Huynh Thuy Phuong, Dong Thi Hoai Tam, Nguyen Minh Nguyet, Trung Dinh The, Thuy Nguyen Thi Van, Hang Nguyen Thi Thu, Kien Vu Duy, Hung Vu Bao, Dung Do Van, Tuan Diep Tran, Bridget Wills, Jennifer Ilo Van Nuil, Evelyne Kestelyn

**Affiliations:** 1 Oxford University Clinical Research Unit Ho Chi Minh City Vietnam; 2 University of Medicine and Pharmacy at Ho Chi Minh City Ho Chi Minh City Vietnam; 3 Centre for Tropical Medicine and Global Health Nuffield Department of Medicine University of Oxford Oxford, England United Kingdom

**Keywords:** medical students, healthy volunteers, research ethics, clinical research, perception, attitude, motivation, recruitment efforts, young generations, Vietnam, mixed methods study

## Abstract

**Background:**

Recruiting and retaining adequate numbers of eligible participants remain the key challenges in clinical research. Understanding the factors associated with participants’ motivations is essential to support recruitment efforts, reduce early withdrawals, and consolidate commitment. The Oxford University Clinical Research Unit conducted a longitudinal study, named the SEED project, with a cohort of first- and third-year students at the University of Medicine and Pharmacy at Ho Chi Minh City, Vietnam.

**Objective:**

This paper describes the findings of the SEED project related to students’ understanding of clinical research and characterizes factors influencing their motivation to participate.

**Methods:**

We used a mixed methods approach, incorporating surveys, in-depth interviews, and focus group discussions to collect insights from students on ethical and practical aspects of clinical research participation.

**Results:**

A total of 437 students were enrolled, with the majority coming from the general medicine faculty. Of these participants, 74 students contributed to qualitative data. Over 95% of the students agreed that clinical research could make an important contribution to science (430/435, 99%) and the health of society by increasing disease awareness (422/436, 97%) and potential access to more effective treatments (415/435, 95%). Few students (81/435, 19%) expressed concerns about the negative impacts of clinical research on the environment. In terms of risk, most students emphasized unpredictable or serious side effects (226/434, 52%) or inconveniences (257/435, 59%) as major concerns, whereas small proportions worried about the risk of disclosure of personal information (94/436, 22%) or the risk of being treated like an “experimental subject, not human being” (33/434, 8%). In in-depth interviews and focus group discussions, health-related benefits, opportunities for intellectual growth, time requirements, and altruistic attitudes built on the perceived social value of clinical research were highlighted as key factors influencing students’ participation.

**Conclusions:**

Students in this study expressed favorable attitudes toward clinical research. By highlighting altruistic motivations built on the perceived social value of clinical research and personal motivations based on perceived health-related benefits for participants, this study provides insights to inform recruitment efforts for clinical studies involving student participants or other young, healthy individuals.

## Introduction

Multiple factors contribute to the success of clinical research, but the ability to recruit and retain adequate numbers of eligible participants is a crucial element and, for many investigators, the most challenging aspect of conducting research [1]. In the context of clinical trials, inadequate enrollment may result in early study terminations or the need for expensive financial and/or time extensions [2]. Similarly, in observational studies, a low participation rate may create nonresponse biases and reduce statistical power, posing a threat to the validity and generalizability of the study findings [3,4]. Understanding the factors associated with participation in clinical research has increasingly been recognized as an essential step to support recruitment efforts, reduce early withdrawals, and consolidate participant commitment [1,5,6].

The existing literature has highlighted a range of determinants that may impact willingness to participate in clinical research, including concerns about potential risks and side effects, limited understanding of the research information presented, mistrust of the research team, time, and location impediments, as well as language, literacy, and other sociocultural factors [5-8]. With respect to study design, high-risk and interventional studies (eg, clinical trials) are associated with higher rates of consent withdrawal and dropout rates, compared to observational studies [9].

Several reports have described participants identified as more likely to be willing to contribute to clinical research as being male [10,11], having the illness under study, or having sick relatives [8,12-14], being middle aged or older people [13,15], having previous experience of participation in clinical research [12,14], or having a generally positive attitude toward participation in clinical research [12,13]. However, other researchers did not identify a significant association between age and willingness to participate in clinical research [11,16,17].

A positive association between having a biomedicine-related degree and willingness to participate in clinical research has been described [10,11,15]. However, despite a desire to contribute to the development of medical science and education [18,19], medical students are sometimes hesitant to participate in clinical research due to time commitment constraints [19]. In addition, concerns about possible coercion by academic tutors involved in the research have been voiced [20,21].

In 2018, we initiated a research project at the Oxford University Clinical Research Unit in Ho Chi Minh City, aiming to engage with a wide range of stakeholders to explore their perceptions and views on clinical research in Vietnam. Involving stakeholders, including funders, policymakers, health practitioners, researchers, and communities, increases legitimacy, credibility, acceptability, and practices aligning with ethical principles in biomedicine research [22]. The first report of our interactions involving senior, national, and international stakeholders was published in 2019 [23]. However, the remit of our project also extended to exploring opinions among younger generations of Vietnamese society, especially students of the health professions, who might conceivably be involved in research in the future, potentially as clinicians, scientists, data collectors, or even as research participants. We conducted a longitudinal study (named the SEED project) involving a cohort of students attending the University of Medicine and Pharmacy (UMP) at Ho Chi Minh City from July 2020 to December 2024. This paper describes the initial findings of the SEED project related to the students’ general perceptions of clinical research and their motivations to participate in such research, aiming to specifically characterize the students’ understanding of the benefits, risks, and burdens of clinical research.

## Methods

The development and overall structure of the SEED cohort have been reported in detail elsewhere [24], but a relevant summary is provided in the Participant Recruitment and Study Procedures section.

### Ethical Considerations

The SEED study was approved by the Oxford Tropical Research Ethics Committee (approval: 540-20—dated July 2020) and the ethical committee of the UMP at Ho Chi Minh City (approval: 351/HDDD-DHYD—dated May 26, 2020). Study staff discussed the study program with potential participants and provided them with a written information sheet, describing the purpose of the study, the procedures, possible risks, benefits, and the rights and responsibilities of participants. Written informed consent was obtained from all students before any study procedures were implemented. Students were compensated for time spent and travel, where applicable. All participants provided written consent for publication of deidentified data. Study staff ensured that all information generated in the SEED study remained confidentially and securely stored. Students’ privacy was protected by deidentifying personal information and replacing their names with confidential participant numbers.

### Participant Recruitment and Study Procedures

Potential participants included first and third-year students attending the faculties of medicine and public health at UMP. Eligible students, who were aged at least 18 years and who expressed willingness to participate in project activities for the next 3 years while studying at UMP, were invited to join the cohort after signing a consent form.

Upon enrollment, students were asked to complete a demographic questionnaire and a comprehensive survey (CS) that included questions about their perceptions and attitudes toward clinical research in general, as well as their thoughts about specific types of research. Subsequently, a variety of topics relating to clinical research in human participants were addressed with students by combining in-depth qualitative methods with synergistic engagement activities. A new topic was introduced every 2-3 months in a sequential manner. We organized a series of engagement activities (such as science cafes, science debates, and role play events) as interactive platforms for the cohort participants to learn basic information about each particular topic. Those events also helped us explore the thoughts and opinions related to the often-complex concepts encountered. Later, among those who had attended a related engagement activity, we purposively selected participants for in-depth interviews (IDIs) and focus group discussions (FGDs), ensuring diverse representation of cohort members based on predefined characteristics, including sex, socioeconomic background, faculty, and academic year of study. In this way, we hoped to ensure that the students attending the FGDs and IDIs were aware of general background information about research and well prepared to discuss the relevant issues surrounding each topic in more depth. The selected students received an invitation via email and were free to choose whether to participate in the IDIs or FGDs.

Here, we report our findings related to the main topics addressed with the cohort participants, focusing on students’ thoughts around the ethics of clinical research generally and also specifically exploring their thoughts on motivations to participate in research. The four main topics relevant to this report were (1) clinical research and how it is relevant in Vietnam, (2) vaccines and vaccine trials, (3) vulnerability, and (4) reimbursement and compensation.

### Data Collection and Analysis

The study used a mixed methods approach using quantitative methods (questionnaires and surveys), which were completed by all SEED cohort participants, together with qualitative data from IDIs and FGDs, which were attended by smaller groups of purposefully selected students.

#### Comprehensive Survey

The self-administered CS (Multimedia Appendix 1) was designed to collect data on students’ attitudes toward benefits, risks, burdens, and motivations regarding clinical research involving human participants. The survey was structured into 4 sections, with the first section focused on exploring their perceptions of clinical research in general, and the subsequent sections specifically exploring their views on observational research, clinical trials, and human challenge studies. For this paper, we report data primarily from the first section. In this section, students rated their level of agreement on a Likert Scale, with several statements on the benefits and risks of clinical research, both for individual study participants and at a community level. In addition, the students were asked to respond to a series of questions about what might influence their own personal decision-making if approached to participate in a clinical research study.

#### FGDs and IDIs

Questionnaire and interview guides were developed based on the published literature, together with feedback on issues raised by students attending the engagement events. The initial structured questions were designed to encourage students to share their personal narratives and thoughts, later supplemented by more probing questions to allow a deeper exploration of their views.

### Data Analysis

All quantitative analyses were performed using R software (R Foundation for Statistical Computing). Descriptive statistics were used to summarize students’ demographic information and perception outcomes: frequency and percentages for categorical variables, mean and range for continuous variables. A 5-level Likert scale (strongly disagree, disagree, neutral, agree, and strongly agree) was used to explore students’ agreement with factors influencing their decision to participate in clinical research. However, several categories in the original 5-level Likert scale contained very small frequencies for multiple items, particularly at the extreme response levels. To avoid sparse-data bias and ensure stable model estimation, we collapsed the categories of responses from 5 to 3 levels (disagree, neutral, and agree) to ensure that the cell sizes were large enough to perform robust analyses [25]. We used chi-square tests to examine differences in responses between year groups and between medicine and public health students and adjusted for multiple testing using the Benjamin and Hochberg correction method. Results were considered statistically significant at a *P* value <.05.

All qualitative data were audio-recorded, transcribed verbatim, and then uploaded into NVivo (version 12; Lumivero) software for management and analysis. Although the data from the FGDs and IDIs were transcribed and analyzed in Vietnamese, we used English codes to ensure accessibility for the full research team. We translated the quotes presented in this paper into English after the analysis was completed. A grounded theory coding approach [26] was used to generate the central ideas for data interpretation and to construct a framework to describe the students’ perceptions, attitudes, and willingness toward clinical research. Two independent Vietnamese researchers (CHL and VP-T) used open-coding techniques (line-by-line coding) to code a subset of FGDs and IDIs, then compared their analytical approaches and discussed overarching categories with English-speaking team members until they reached consensus on initial codes. Following these discussions, the refined codebook was applied to the full dataset, with ongoing disagreements resolved through consensus meetings during the analysis process. We then conducted 4 additional IDIs to confirm the initial findings. Subsequently, the study team reviewed all the transcripts and codes and refined and linked categories and subcategories into themes. Within the scope of this paper, we report the themes describing the students’ perception of clinical research’s benefits, risks, and burdens and other factors influencing their motivations to participate.

## Results

### Overview

**Figure 1:**
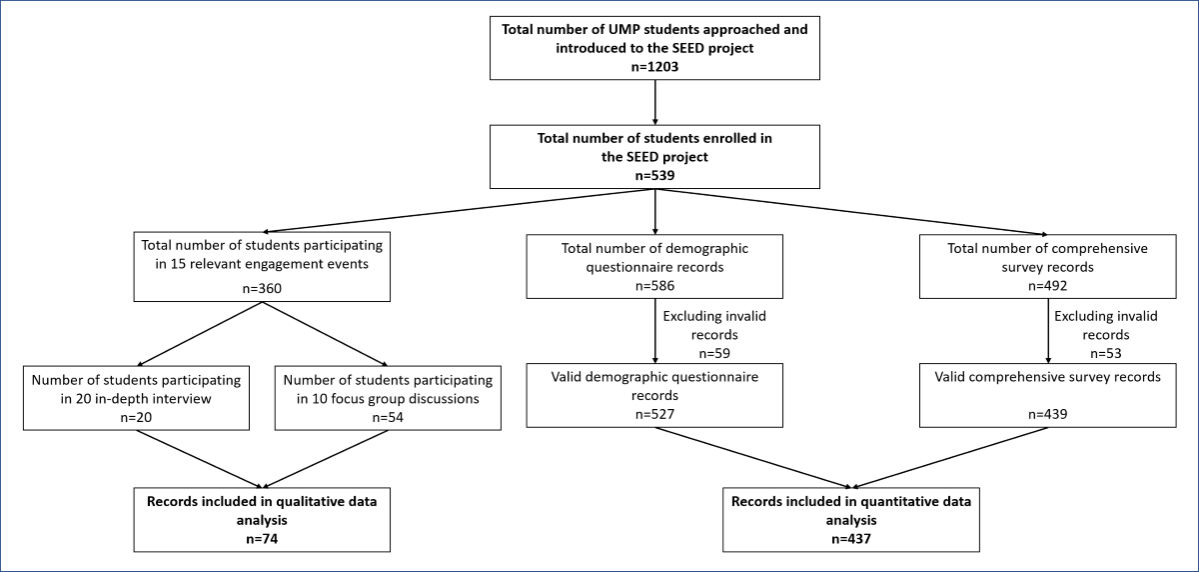
Flowchart of participant activities and data collection. UMP: University of Medicine and Pharmacy. The number in each box represents the count of students or records at each stage.

A total of 1203 UMP students attended one of our introductory events and completed introductory forms between July 2020 and December 2021, during which they were invited to participate in the SEED project (Figure 1). Among this group, 539 students enrolled in the study and were eligible to contribute to the various activities until March 2023; individual students attended variable numbers of events ranging from 0 to 6, depending on the timing of their recruitment to the cohort. After removing duplicate or invalid records (due to lack of study code or no consent documented), the final study population for this analysis comprised 437 of 539 students with demographic data available, who had completed the initial CS. Among these individuals, 336 of 437 (77%) students attended at least 1 relevant engagement activity related to the 4 topics of interest. A total of 10 FGDs and 20 IDIs were conducted in parallel with these activities, with 74 students participating in either an IDI (n=20) or an FGD (n=54), thereby providing qualitative as well as quantitative data on the study topics (Figure 1).

### Demographic Characteristics of the Analysis Population

Demographic characteristics of the 437 participants included in this analysis are presented in Table 1. The majority were students of medicine (302/437, 69%), with lower representation from the preventive medicine (63/437, 14%) and public health (45/437, 10%) departments, and only a few students (27/437, 6%) participating from the nutrition department. A little over half the students (254/437, 58%) were enrolled in their first year. Although male and female participants were equally represented in the cohort overall, breakdown by faculty indicated that 64% (192/302) of medical students were male, while 79% (106/135) of public health students were female. Almost all students were ethnic Kinh (392/437, 90%), in line with the expected proportion across the Vietnamese population [27].

**Table 1:** Demographic characteristics of SEED cohort students included in this analysis.

		Faculty of General Medicine	Faculty of Public Health^a^				Total (N=437)
		General medicine (n=302, 69%)	Preventive medicine (n=63, 14%)	Public health (n=45, 10%)	Nutrition (n=27, 6%)		
**Age (years)**							
	Mean (SD)	19 (1)	20 (2)	20 (1)	19 (1)	19 (1)	
	Range	18-23	18-25	19-22	19-21	18-25	
**Sex, n (%)**							
	Male	192 (64)	12 (19)	15 (33)	2 (7)	221 (51)	
	Female	110 (36)	51 (81)	30 (67)	25 (94)	216 (49)	
**Academic year at enrollment, n (%)**							
	First year	169 (56)	34 (54)	29 (64)	22 (81)	254 (58)	
	Third year	133 (44)	29 (46)	16 (36)	5 (19)	183 (42)	
**Ethnicity^b^, n (%)**							
	Kinh	269 (90)	59 (94)	40 (89)	24 (89)	392 (90)	
	Hoa	17 (5)	1 (1)	2 (4)	2 (7)	22 (5)	
	Others	14 (5)	3 (5)	3 (7)	1 (4)	21 (5)	
**Family monthly income^b,c^, n (%)**							
	Less than 3 million VND	4 (1)	3 (4)	1 (2)	0 (0)	8 (2)	
	3-10 million VND	62 (22)	17 (27)	14 (34)	7 (27)	100 (24)	
	10-60 million VND	118 (40)	18 (29)	13 (32)	6 (23)	155 (37)	
	Over 60 million VND	6 (2)	1 (2)	1 (2)	1 (3)	9 (2)	
	Do not know	64 (22)	12 (19)	7 (16)	9 (35)	92 (22)	
	Prefer not to answer	39 (13)	12 (19)	5 (12)	3 (12)	59 (13)	
**Socioeconomic status^b,d^, n (%)**							
	Poor	18 (6)	5 (8)	2 (5)	2 (7)	27 (6)	
	Average	244 (83)	49 (79)	33 (77)	20 (74)	346 (82)	
	Wealthy	0 (0)	1 (2)	1 (2)	0 (0)	2 (0)	
	Prefer not to answer	33 (11)	7 (11)	7 (16)	5 (19)	52 (12)	

^a^Public health faculty includes the departments of preventive medicine, public health, and nutrition.

^b^Missing data: for ethnicity=2; for family monthly income=14; and for socioeconomic status=10.

^c^VND refers to Vietnamese Dong. 1 VND=US $0.00004328 (average VND to US dollar exchange rate in December 2020). For the family income bands, we applied the Vietnamese government’s poverty threshold for 2016-2020 [28], and the 21-time disparity in income between the poorest and richest populations identified by Oxfam in their 2017 survey [29].

^d^Socioeconomic status refers to the students’ personal assessment of their family’s overall socioeconomic status within the Vietnamese context.

Most students (255/423, 61%) reported that their family’s monthly income fell within the range of 3 to 60 million VND (approximately equivalent to US $121-$2432 per month, generally considered as “average”). Only a few students (17/423, 4%) stated that their families earned more or less than this range, and 36% (151/423) of the students responded “do not know” or “prefer not to answer” to the income questions. When asked to subjectively characterize their personal circumstances in the Vietnamese context, 346 of 437 (81%) students categorized their families’ overall socioeconomic status as average, while a small proportion of students (27/427, 6%) stated their family as poor, the rest preferred not to respond.

The 437 SEED cohort participants included in this analysis were reasonably representative of the eligible student population, although with a significantly greater proportion of public health students involved (Table S1 in Multimedia Appendix 2). A total of 74 students with diverse demographic characteristics were purposively selected to participate in the FGDs or IDIs (Table S2 in Multimedia Appendix 2).

**Figure 2:**
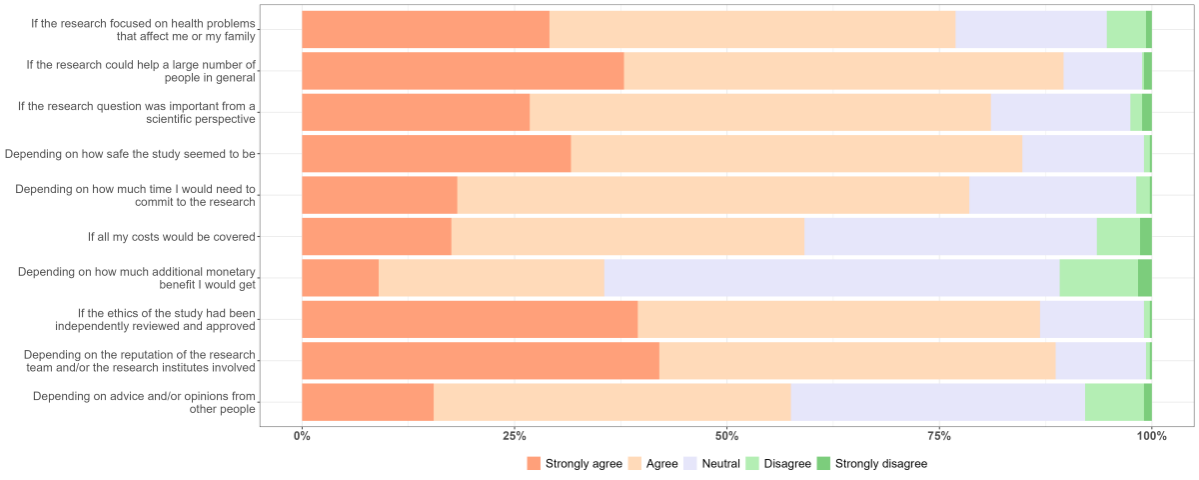
Factors influencing students’ motivation to participate in clinical research.

**Figure 3:**
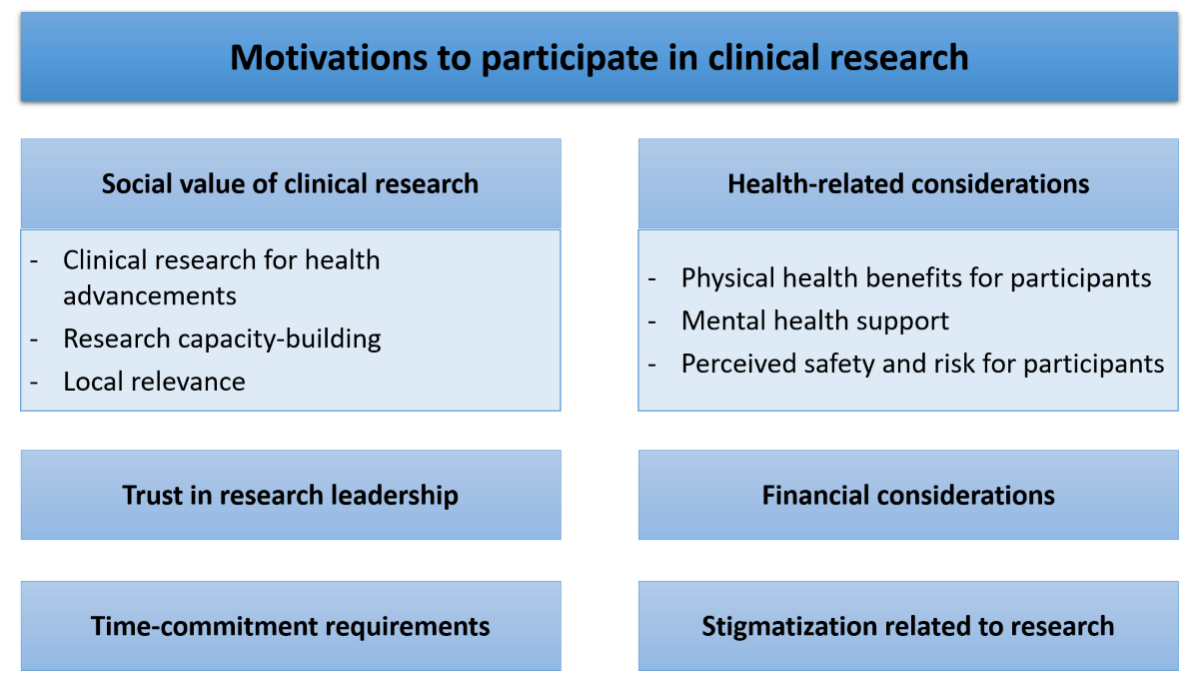
A framework for motivation factors of clinical research participation.

Overall, students’ decision-making about whether to participate in clinical research was often shaped by their perception of benefits, risks, and burdens associated with the studies, highlighting students’ recognition of impacts on general society and individual participants. Most students agreed that the factors mentioned in Figure 2 might influence their own decision to participate in a clinical research study, except for the promise of additional monetary benefits. When asked to identify the most important factors influencing their decision-making to participate in clinical research, they primarily selected the relevance of research objectives to their own health or their family members’ health, the potential for the study to benefit a large number of people, and to what extent the study appeared safe, followed by considerations relating to ethics approvals and the reputation of the institutions involved (Figure S1 in Multimedia Appendix 2). This section presents the quantitative and qualitative data analysis of students’ perceptions on benefits, risks, and burdens of clinical research and how these perceptions influence their decision-making to participate (Figure 3).

### Motivation Driven by Perceived Social Value of Clinical Research

#### Clinical Research for Health Care Advancements

**Figure 4:**
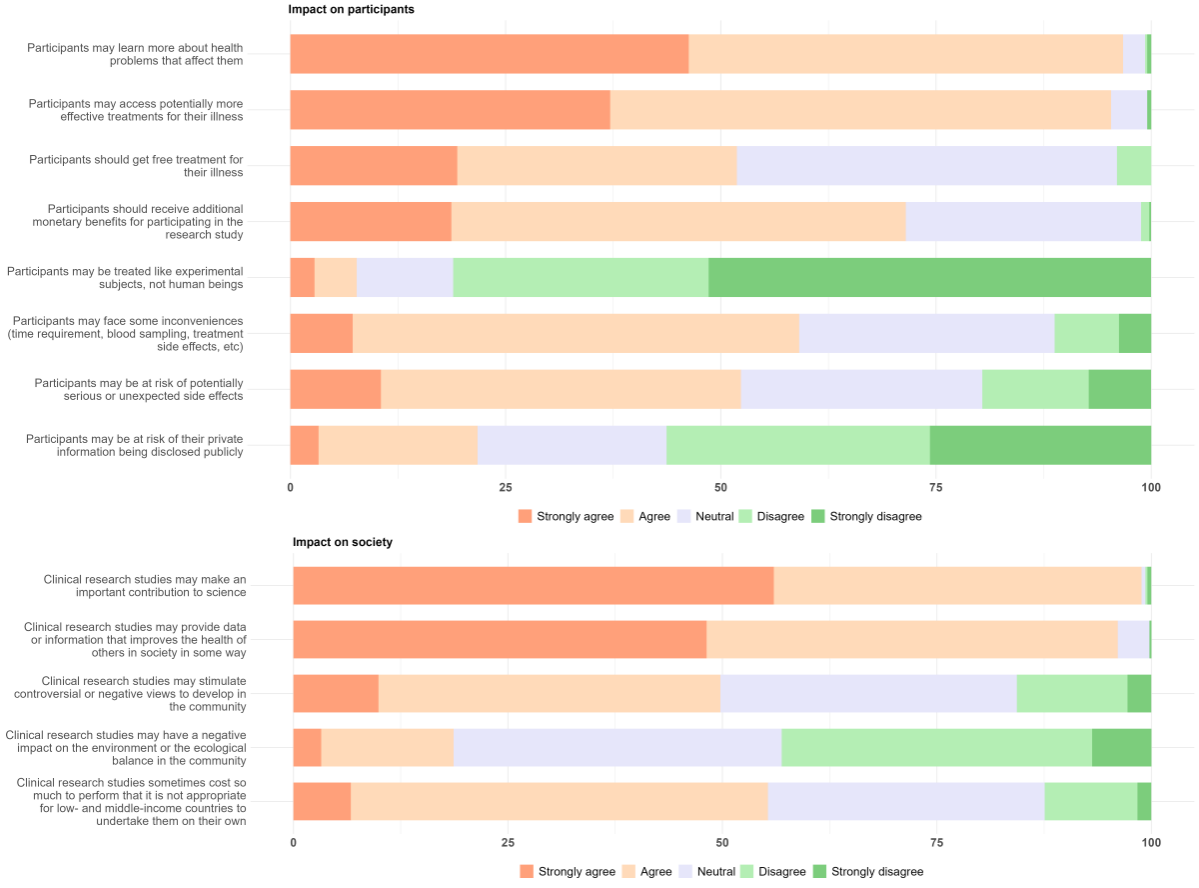
Students’ perceptions of the potential impact of clinical research on society and participants.

The data from the CS showed that almost all students (430/435, 99%) agreed that clinical research could make an important contribution to science and that data or information from research could help improve health in society (418/435, 96%; Figure 4). The perception that clinical research could benefit a large number of people was also identified as one of the most important factors influencing students’ motivation to participate in clinical research (Figure S1 in Multimedia Appendix 2). In the IDIs and FGDs, students also emphasized how contributing to scientific development was one of the most important outcomes of clinical studies. A crucial principle of research, in their opinion, should be knowledge generation, leading to improvements in medical care or the development of novel public health interventions. Such advances were likely, in turn, to increase public trust in health systems. Students also noted that research conducted in high-prevalence settings could provide a better scientific foundation to explore disease causation and develop treatment strategies appropriate to the local epidemiological characteristics and context. These types of foundations would be directly beneficial to the people who carry the burden of such diseases. Improvements in disease control were another valuable outcome or impact of clinical research mentioned by the students. For instance, local vaccine-related clinical research efforts might result in reduced dependence on external vaccine supplies and greater autonomy in domestic vaccine development and manufacture, which could lead to broader vaccine access for the population, as well as contribute to greater public awareness of the benefits of vaccination. The students also noted that clinical research efforts could impact national solidarity by promoting altruistic values and encouraging community support.

I think the government provides partial funds for vaccine research. But in addition to government funding resources, there is great support from big corporations or celebrities [public figures in the countries] aiming to buy vaccines or develop domestic vaccines. This creates positive effects [in society] and at the same time enhances national unity, helping us to actively control the disease situation.

FGD4 on vaccines and vaccine trials

Conversely, alongside the mission of knowledge advancements, more than half the students (240/435, 55%) had concerns about the need for substantial investment in clinical research, such that it may be inappropriate for low- and middle-income countries to undertake such studies on their own. In the FGDs and IDIs, they explained that government resources should be preferentially allocated to address major public health issues (eg, lack of clean water, air pollution, or hygiene and sanitation) or to enhance health infrastructure and facilities, rather than investing in clinical research, which might be perceived as time-consuming with uncertain benefits.

#### Research Capacity-Building

Some students expressed the view that the success of specific research would depend on its overall social value for the community, whereas “research failure” would be determined not only by the occurrence of adverse effects on the health of study participants but also by other factors such as not following credible research procedures and lack of adherence to ethical principles. In the case of “unsuccessful research,” which the students described as when research findings differ from the investigators’ initial hypothesis, they believed that the research could still be valuable, for example, by improving research capacity during the establishment of a clinical study.

In my opinion, I think research only fails when it is done by using the wrong process. Even if the results are not as expected, the research still gives you lessons; it teaches you research methods. As for the results, I think that no matter what the result is, it is still a success, to a greater or lesser extent.

FGD2 on clinical research and how it is relevant in Vietnam

#### Local Relevance and Personal Motivation

Regarding the perceived social value of clinical research, a high percentage of students emphasized that they would consider participating if the research could help a large number of people (392/347, 90%) or because of the scientific importance of the research questions (352/435, 80%; Figure 2). In the IDIs and FGDs, they emphasized that they would consider the epidemiology of the disease before making the decision to participate. For instance, they would consider participating in a vaccine trial if the incidence rate of the disease being studied was high in their region and if it was challenging to access vaccines (eg, at the beginning of the COVID-19 pandemic). Otherwise, they would not participate in a vaccine trial if the disease was not prevalent in Vietnam.

When I participate [in a vaccine trial], I want to gain some benefit for myself. It could be to create an immune response against the disease. But if the disease is not urgent enough to worry me, I will not join.

FGD5 about vaccine and vaccine trials

Survey data showed that 77% (336/437) of students indicated that they would participate in clinical research that addresses health problems affecting themselves or their families (Figure 2). Although in the unadjusted analysis, slightly more students enrolled in the medicine than the public health faculty indicated that they would participate if the research focused on health problems relevant to them or their families (*P*=.04, chi-square test); no significant differences were apparent between the faculties after adjustment for multiple testing (Table S3 in Multimedia Appendix 2). This reflected the students’ instrumental motivation, as they believed that their participation could benefit their family members’ health by contributing to the ongoing development of relevant investigational products as potential therapeutics, which in turn would benefit society.

That is the community-driven mindset of each person, [desire] to contribute something to the community. Then it also a contribution to myself.

FGD2 on clinical research and how it is relevant in Vietnam

Interestingly, students were personally motivated to participate in clinical research by intellectual advantages, including the potential to gain new knowledge and experiences that could be valuable for their future careers, especially in the field of biomedical research.

### Health-Related Considerations

#### Physical Health Benefits for Participants

Most students agreed that participants in clinical research could learn more about their disease (422/436, 97%) or access potentially more effective treatments for their illness (415/435, 95%; Figure 4). Additionally, several health-related advantages of clinical research in terms of participants’ physical and mental health were mentioned by students in the FGDs and IDIs. First, the students highlighted the possibility of disease remission or cure through access to new drugs or new treatment strategies being applied in a research context, especially if they were experiencing serious illnesses without alternative treatments (eg, cancer).

Cancer patients, for example, if they are in the final stage [of the disease] and there is a study about something like a treatment measure that can improve their cancer condition, they might think, “oh I will die anyway, so I could try it.”

IDI10, nutrition, third year, female student

Students also commented on the potential value of clinical research for vulnerable populations (eg, children, pregnant women, and older people) by providing them with access, under carefully supervised, controlled conditions, to new health care products, treatments, and services that are not typically recommended for them or in tailoring these products specifically for their needs.

I think vulnerable groups should be involved in [clinical research]. They should be allowed to participate equally in research compared to other people. Currently, only a limited number of research studies involve vulnerable groups such as children or pregnant women, yet these groups need more attention and care than others.

IDI10, nutrition, third year, female student

Personally, the students highlighted that their health conditions could be improved through access to free health check-ups before, during, and after the clinical research process. Further, they thought that they might develop immunity to the disease being studied through participation in vaccine trials, as one student mentioned:

If it [the trial vaccine] works, I will be among the first ones having immunity against that virus, won’t I? I mean, I will be the one to get protection first.

IDI4, medicine faculty, first year, female student

#### Mental Health Support

A second dimension of health-related benefits for study participants mentioned by the students was mental health support. They included the possibility of developing supportive networks among participants during the clinical research process, encouraging informational and emotional sharing opportunities, as well as providing specific mental health counseling services (eg, stress relief) by the investigators.

I think it’s possible to create a network with study participants. We have connections and bond them together. Even though the research is over, they [study participants] can still be in contact and help each other ... Some people join the study because they want to express and share their feelings [with other patients] or find solutions for their problems.

IDI3, medicine faculty, third-year, male student

#### Perceived Safety and Risks of Participation in Clinical Research

As shown in Figure 4, half of the students believed that participants in clinical research might be at risk of unpredictable or serious side effects (226/434, 52%), whereas few students worried about the risk of being treated like experimental subjects (33/434, 8%). In the FGDs and IDIs, students also elaborated on potential risks for participants’ physical and mental health. For example, anaphylactic reactions, death, and disability were serious physical health consequences noted by the students; some students emphasized that they would refuse to participate in a clinical study if any of these significant health issues were listed in the informed consent, regardless of the likelihood of such events occurring. Others, however, considered the potential value to the community and weighed the severity of the disease (based on incidence and mortality rates) against the risk of participation.

If people [investigators] explain that a study, for example, its risks [for participants] could be fatal, or could result in disability or paralysis, I feel it’s dangerous. I will worry. So, I will not participate. On the other hand, [if the study has] some mild complications, or the incidence rate of severe complication is very low, and its [the study] benefits are great, I will participate.

IDI3, medicine faculty, third-year, male student

In terms of side effects, students indicated that they would accept research with temporary or mild side effects, such as fever, pain, or swelling at injection sites.

I think the side effects are acceptable if, at a minimum, they won’t affect my daily life activities later on. These effects might last a few hours or a few days, but in the future, they might not have much impact. It’s okay. These side effects are acceptable.

FGD3 on vaccines and vaccine trials

Other concerns revealed by the students in the IDIs and FGDs focused on the investigational products (eg, drugs or vaccine candidates or other interventions) and the study methodologies used in clinical research. The students indicated that they might have a limited understanding of the origin of investigational products and how the research methods were to be applied to estimate and control the interactions of these products with their intended biological targets. This, in turn, could contribute to students’ concerns that the investigators might not be able to fully anticipate potential adverse effects on participants’ health, leading to reservations about safety or unexpected health issues arising, not only during a study but also in the poststudy period or after completion of the designated follow-up visits. Notably, some students said that they would prefer to participate in studies using investigational products manufactured in high-income countries rather than those developed domestically because of a presumed higher level of manufacturing quality. Additionally, they indicated concerns about access to study-related health benefits, particularly access to the intervention being evaluated, among participants allocated to the control group in placebo-controlled studies. Regarding methodology, the students believed that, although findings from previous studies could provide a useful foundation for ongoing research efforts, using a design previously conducted in a study population with different genetic and epidemiological characteristics could lead to unexpected health consequences. Thus, assessing a vaccine previously tested elsewhere, in a new population, might result in unforeseen adverse effects—for instance, through interactions with other vaccines already deployed in the new setting or due to major differences in preexisting immunological profiles in the new study participants compared to the original group.

For example, talking about a trial conducted in America. These people, their physical health, their awareness of health, and the health system are different [to the Vietnamese context]. Their bodies are not exposed to as many infectious diseases as ours [Vietnamese people]. And their body sizes are also larger, and they might have better immunity. If we do the same trial here, the results may be different.

FGD3 on vaccines and vaccine trials

### Participation Decision Fostered by Trust in Research Leaderships

Data from CS showed that approximately 90% (388/437) of students linked their decision to participate in clinical research to the capacity of the research institutions involved (Figure 2). In the IDIs and FGDs, the students preferred studies conducted by institutions with “good reputations,” which they assessed based on the number of studies conducted previously, the degree qualifications of the research staff, and collaboration with government agencies such as public hospitals, universities, or the Ministry of Health. Through such partnerships with government agencies, the students believed that the studies were approved ethically and legally, the participants’ rights were secure, and compensation was available for any study-related harm. However, some students were still worried about rigorous adherence to safety principles during the research process and about the capacity of the research team and hospital facilities to respond effectively to study-related adverse events. In addition, trust in institutions might be built based on whether the study provided benefits to participants.

If research provides remuneration, I feel like that research group is more professional because it has a part to return to the participants what the participants lost. The group carefully calculates the portion that the participant loses, so it will reimburse the exact portion that the participant has lost. And if it’s not all about money, but medical support for participants, that’s also a form of compensation.

... For research that has much impact on human health, by so if ensuring people’s safety is given more priority, the research group will be [considered] more professional. As for research on the use of drugs, it has little impact on human safety [e.g., lab-based studies], [providing] more money [to participants] is ... I think it’s more professional.

IDI16, public health faculty, female, third-year student

In addition, relatively few students (81/435, 19%) worried about potential negative impacts of clinical research on the environment or the ecological balance in the community, although 38% (166/435) of students gave a neutral response (Figure 3). Regarding these attitudes, students explained in the FGDs and IDIs that they believed that the investigators, under the supervision of ethics committees and other government agencies, were obliged to set up procedures to manage research waste and prevent pathogen transmission to the community prior to and throughout the research process.

### Financial Considerations

Monetary factors were discussed, but their impacts on the students’ willingness to participate in clinical research were minor. Data from CS showed that over half of the students (260/437, 59%) reported that they would participate in clinical research if all their costs were covered, with a higher preference among first-year students than third-year students (*P*=.03; Table S3 in Multimedia Appendix 2). A minority of them (156/437, 36%) considered the additional monetary benefits they would get (Figure 2). In the IDIs and FGDs, students elaborated on the impacts of financial considerations on their willingness to participate in clinical research, in terms of reimbursement, compensation, and incentives.

Students pointed out that out-of-pocket expenses associated with participating in a research study (eg, transportation or accommodation expenses) might be a financial barrier for some people, especially those with low incomes. Reimbursement, considered in this study context as money given to the participant to refund their expenses, could encourage people to participate in clinical research. However, the students also conveyed that reimbursements would not be a major influence on their own decision to participate, especially if the study offered health-related benefits. For instance, study-related health benefits such as free access to new vaccines or treatments could motivate them to overcome any inconveniences, even if their expenses were not covered. Incentives, defined here as gifts (ie, money or material items) used to encourage people to participate and commit to research, could be another financial motive. However, students also noted that such incentives would likely have little impact on their own decisions on whether to participate in a study. They might be attracted by incentives, but only for low-risk studies (eg, observational studies) or studies with no health-related benefits. Compensation, the final dimension of remuneration that students discussed in the IDIs and FGDs, was defined as money or items given to participants to acknowledge time spent, data and specimen contributions, or other practical efforts they provided to support the clinical research.

When people participate in a study, they must put in some kind of effort. So ... generally, we [the study team] should give people something in return, so that they feel what they put in is worth [their time].

FGD10 about reimbursement and compensation

Further, students emphasized the importance of having a mechanism to compensate for unanticipated losses and harms related to the study. This might take the form of health insurance or financial restitution for future consequences of clinical research, such as physical and mental health impairment or disclosure of personal information. They perceived insurance as a right to which all participants should be entitled and as an essential component that should be compulsorily implemented in every study protocol. The provision of a financial support structure was vital to their decision-making to participate in clinical research because it would demonstrate the research team’s commitment to addressing the future risks and preventing participants from encountering significant or even catastrophic out-of-pocket health expenditures after completion of the study.

Economic burden ... Supposing that I am the breadwinner of the family, and I have a health problem [as a result of the study], then I will become a burden to my family ... Most studies, from what I read, do not show their commitment to support the [participants’] family if something wrong happens, therefore, I think the family will be the first to suffer. And it also causes anxiety for society and for people around me.

IDI4, medicine faculty, first-year, female student

### Perceived Burden and Inconveniences of Clinical Research

#### Stigmatizations Related to Clinical Research

As shown in Figure 4, half of the students believed that participants in clinical research might be at risk of inconveniences (257/435, 59%), whereas a few students worried about the risk of public disclosure of personal information (94/436, 22%). In the FGDs and IDIs, the students explained that they would not worry about confidentiality because they trusted the ethics committees to supervise data security and ensure that the study team rigorously followed data protection regulations. However, some students commented that participation in certain types of research could make them become a source of onward transmission to third parties (eg, family members or community contacts), and that, in turn, this could result in them experiencing social stigmatization. Furthermore, negative consequences on their physical health, especially severe adverse events, could lead to emotional and financial distress for study participants and their families.

#### Time-Commitment Requirements

Over 70% (334/437) of students agreed that estimations of time demands were essential for their considerations of participating in clinical research (Figure 2). The required time had more impact on third-year students than first-year students, as shown in both unadjusted (*P*=.001) and adjusted analyses (*P*=.01, chi-square test; Table S3 in Multimedia Appendix 2). During the IDIs and FDGs, the students noted that the main burdens making them hesitant to participate in clinical research could be the amount of time they would be required to commit to the study and its potential impact on their academic studies. As health care students often had hectic schedules, they found it difficult to commit to a study requiring a great deal of time. Thus, they worried that participation in clinical research might disrupt their academic studies as well as their daily lives, especially a research project requiring them to be absent from college.

I think that if I participate in research, I must invest a lot of time in it. But as a student, I feel that I do not have enough time to participate in such studies.

IDI1, medicine faculty, first-year, male student

## Discussion

### Principal Findings

Clinical research was held in high esteem by students, who expressed positive views of a broad range of societal and personal benefits. The main value of clinical research highlighted by the students was scientific advancement leading to enhanced community health and active disease control. This perceived worth increased their altruistic motivation to participate in clinical research significantly. Personal benefits, such as access to free health care services and opportunities for advanced medical education, were linked to the students’ health-related and intellectual motivations.

Although monetary considerations were discussed throughout the FGDs and IDIs, students might not view them as the primary reason supporting their decision to participate in clinical research. This result is comparable to a study conducted by Soule et al [30] examining participants’ motivation to enroll in nontreatment-based research studies, in which they found that altruism had a more significant impact on participants’ motivation for study involvement than health care or financial benefits. Additionally, given that the majority of students in the cohort considered themselves young and healthy people, their motivations to participate in clinical research using invasive procedures or investigational products are similar to those found in empirical studies on healthy volunteers in clinical research [31,32]. Manton et al [31] found that the prospect of self-development in health science, comprising the gains of valuable learning, life experiences, and opportunities to increase social interactions, could significantly promote healthy people to participate in clinical research. This idea was also brought up when we inquired from the students about their intellectual motivation to participate in our cohort study. The students’ intellectual motivation also included curiosity about the scientific rationale behind clinical research, reinforcing their desire to contribute to research to advance future health care options. Therefore, it would be worthwhile to investigate the possibility of a connection between intellectual and altruistic motivations in the future.

The second noteworthy factor motivating students to participate in clinical research, according to our study, is their own self-interest in the positive effects clinical research could have on the community and their own health. This finding is consistent with a variety of literature reporting healthy participants’ motivations for clinical trials including altruism, receiving free examinations, access to new advanced preventive treatments, or obtaining money [31-35]. Further, in previous research investigating 4 different forms of contingent relationships between participants’ self-interest and altruism, Olsen et al [36] found a large group of people whose participation decision was motivated by altruism but also prioritized avoiding harm. Similarly, in our study, students reported that they would be willing to participate in clinical research for the sake of others but had safety limits to their altruism [36]. Thus, investigators should be aware of the complexity of the motivations during informed consent sessions, clarifying unreasonable expectations of therapeutic benefits, and encouraging altruistic motives involving the desire to help others without expecting personal gains, especially when recruiting healthy volunteers.

The study findings show that approval of ethics committees and the reputation of research institutions, which were also important to students’ consideration to participate in clinical research, could be interpreted as a form of trust. In this study, only a few students expressed concerns that participants in clinical research might be at risk of being treated as “experimental subjects, not human beings”—that is, that their personal autonomy, rights, and dignity might be compromised during their participation in clinical research. This perspective indicated their acknowledgment of the ethics committees’ roles in scrutinizing clinical research and protecting participants’ rights. It has been documented in previous research that trust contributes significantly to willingness to participate in clinical research [33,37]. Further, participants’ trust in institutions is closely linked to the institutions’ research ethics mechanisms and reputation for integrity [37]. Thus, in order to build participants’ trust, researchers and institutions should develop and maintain their ethics governance systems to acknowledge and transparently follow ethical guidelines and regulations aligned with the local and international principles [37]. Besides the impacts of institutions on participants’ trust, the interpersonal trust between researchers and participants is crucial [11,33,38]. Researchers should consider reframing the participant information sheets and informed consent forms as “trust contracts” that explicitly address what researchers and institutions promise participants to do and not do, in addition to providing crucial research information (eg, research benefits, risks, and requirements). This form of consent could help to foster trust between participants and research teams and enhance motivation to participate in research [37,38].

Contrary to our expectations, we did not find any significant association between the students’ characteristics (except academic year) and their attitudes toward various factors affecting their motivation to participate in clinical research. This finding does not support previous research, in which personal factors such as sex, age, health status, and prior clinical research experience were primary factors influencing decision-making regarding clinical research participation [11,17,39]. A possible explanation could be the differences in study population between our cohort—students from UMP at Ho Chi Minh City, who were generally considered themselves young and healthy, and the other study populations that included members of the general public across various age ranges.

Our study findings indicate that a crucial consideration contributing to decision-making around participation in clinical research was whether the research topics might be relevant to the respondents’ own health issues or those of their family members. This finding seems to be consistent with previous studies showing that health issues of participants or their family members had major impacts on decisions to participate in clinical research [13,39].

### Limitations

This study has some limitations. First, in the SEED project, we initially approached 1203 students registered at the faculties of medicine and public health at UMP to present a brief general study introduction, later providing more detailed information to students who expressed interest, and eventually recruiting a subgroup of 539 students to join the cohort (Figure 1). Despite the similarity in terms of sex balance and academic year distribution, a small difference was apparent in faculty distribution between students in our cohort and those we approached in the original series of introductory talks (Table S1 in Multimedia Appendix 2). This difference suggested that students from the medicine and public health faculties might have varying levels of self-efficacy to manage the demand of cohort activities. Additionally, since we only approached medicine and public health students to introduce the project, the cohort did not include representation from other disciplines such as nursing, medical technology, pharmacy, or traditional medicine. Thus, it is possible that data may not reflect the majority of students from other faculties in UMP. Second, during the ethics review process, the UMP ethics committee was concerned that students might feel coerced to participate in the cohort activities if it could influence their year-end average scores. To mitigate this risk, we clearly assured students in both verbal and written forms that their participation was entirely voluntary and that UMP staff had neither access to the participant list nor involvement in organizing any of the activities. A separate publication is in preparation, describing the students’ experience of participating in the SEED project, but informal feedback indicates that coercion was not an issue for them. Third, although a broad overview of clinical research concepts was given to the students, we did not provide them a detailed protocol of a study, including specifics surrounding diseases, study design and methodology, investigational products, levels of invasiveness, and safety controls. Lack of access to detailed study information could lead to discrepancies between our findings and the actual enrollment in clinical research, especially in clinical trials [40,41]. Future studies should examine the specific circumstances under which behavioral intentions could predict actual enrollment and those in which participation intentions do translate into actions. Finally, we did not explore the impact of COVID-19 on the students’ views about willingness to participate in clinical research. The data collection for this study started in mid-2020 when the COVID-19 pandemic was effectively controlled in Vietnam, with no community outbreaks but only occasional travel related to infections and vaccines not yet available. At that time, it was decided to not include any COVID-19–related questions. In retrospect, this would have been valuable data to collect, but at the time, we could not predict the magnitude or impact of this pandemic. Thus, to preserve the uniformity of the dataset, data on the impact of the COVID-19 pandemic on students’ perception and attitude toward clinical research were not collected, even after COVID-19 became widespread and the Vietnamese government strongly encouraged people with medical backgrounds to volunteer for disease control activities [42]. Many students in our cohort responded to these governmental calls and took part in COVID-19 frontline prevention activities, such as performing diagnostic tests, helping medical professionals treat patients with COVID-19 at hospitals or health centers, or providing information about COVID-19 via free hotlines. Recent studies emphasized the impacts of the COVID-19 pandemic on clinical research recruitment and retention due to heightened anxieties toward clinical research participation, assess barriers, and safety concerns [43,44]. Therefore, we acknowledged that the students’ perceived risk of COVID-19 and their experiences during the COVID-19 pandemic could have impacted their attitudes toward clinical research. These findings underscore the need for further studies to explore the students’ motivation to participate in clinical research within the pandemic context.

### Conclusions

This study found that the majority of students expressed favorable attitudes toward participation in clinical research. Their decision-making was significantly influenced by health-related benefits to themselves or family members, the possibility of intellectual growth, and the time commitment required by the study. The altruistic attitudes rooted in their perceived social value of clinical research also partly encouraged their participation in clinical research. These findings might be helpful for clinical researchers’ understanding when developing outreach recruitment strategies for clinical studies involving student-participants or other young and healthy participants.

## Appendix

Multimedia Appendix 1 Comprehensive survey.

Multimedia Appendix 2 Additional tables and figures. Table S1: Demographic characteristics of SEED cohort participants included in this analysis compared to those of students initially invited to join the cohort. Table S2: Characteristics of students participating in IDIs and FGDs. Table S3: Factors influencing students’ decision to participate in clinical research by faculty and academic year at enrolment (n=437) Figure S1: The most important factors influencing students’ motivation/willingness to participate in clinical research.

## Abbreviations

CS: comprehensive survey
FGD: focus group discussion
IDI: in-depth interview
UMP: University of Medicine and Pharmacy

## Data Availability

The datasets generated or analyzed during this study are available from the corresponding author on reasonable request.
