# Supplementary material for: Vietnamese University Students’ Perceptions and Attitudes Toward Participation in Clinical Research: Mixed Methods Study
Source: J Particip Med. 2026 Feb 12;18:e86269. doi: 10.2196/86269 (PMC12946779; doi:10.2196/86269)
Supplement: Multimedia Appendix 1 [file jopm_v18i1e86269_app1.docx]

**Introduction**

You are invited to complete this survey as you are a participant in the UMP/OUCRU student cohort SEED Project. This is one of several different activities involved in the SEED Project. The overall aim of the project is to explore the knowledge and perceptions of UMP students about clinical research involving human subjects, and to understand the social and cultural factors contributing to these views.

There are many different kinds of clinical research and particular study designs that may have different impacts on aspects of individual participants’ health and life, as well as more broadly on the community. As a result, you may have different ideas and opinions about these different kinds of research – for example you may think that some research studies are ethically acceptable but that others are too risky to be permitted. So, the survey includes several sections in which similar questions are asked about different types of research. In this way we hope to get a comprehensive overview of what you and other UMP students think about the full range of clinical research studies. However, it is important that you understand that we are trying to understand your own personal opinions on these topics, not to test any information you may have been taught at school or university. The survey is completely confidential, your lecturers and tutors are not involved with this project at all, and there is absolutely no link with any assessments you may have at the university or anywhere else.

The most important thing for you to remember is that **there are no right or wrong answers**, and your personal ideas and opinions are very valuable for us. Your contribution will help researchers understand public perceptions and possible barriers to acceptance of clinical research, and also ensure that when new research studies are designed these opinions are properly considered.

Your ideas and opinions may change over time, so we may ask you to complete this survey again at intervals over the next few years while you remain a member of the SEED Project.

Please try to answer honestly what you think. If you really have no opinion about a particular question, then tick the “Neutral” option box. But you can also choose to skip any question or stop doing the survey at any time if you wish.

**Structure of the survey**

The survey consists of 34 questions structured into 4 sections and should take you approximately 30 minutes to complete. Section 1 focuses on your perceptions of clinical research in general, while the other sections will ask more specifically about your views on observational research (section 2), clinical trials (section 3), and human challenge trials (section 4). Each of these sections starts with a short description of that particular type of research.

**Data handling and confidentiality**

All data collected will be used solely for research purposes. The University of Oxford is responsible for ensuring the safe and proper use of any personal information you provide. Information collected from you during the study may be made available to others in the future (for research purposes) provided no one can identify you from the details provided.

If you have any questions or concerns, please send email to chilp@oucru.org or thuyntv@oucru.org (SEED Project Coordinator).

1. **I confirm that I have read and understood the information above, and I agree to participate in this survey**

O YES

1. Date of completion: (dd/mm/yyyy)
2. Participant’s ID number: ……………………………….

**SECTION 1**

This section explores your thoughts and ideas about **clinical research in general** and covers all research involving human participants that is intended to produce knowledge valuable for a) understanding human disease, b) preventing, diagnosing and treating illness, and c) promoting health. Clinical research includes a range of studies involving direct interactions with people (both healthy individuals and people with diseases), biological samples (e.g. blood, urine, tissues specimens), and/or data about different groups of people in society. Some clinical research studies involve deliberately giving some participants specific treatments or interventions and measuring the effects of these treatments, but in other studies the focus is on observing what happens without changing anything from usual practice.

1. What are the potential benefits and risks or burdens of clinical research involving human participants, considered from the perspective of the participants themselves? For each question, please tick the box to choose the option that best represents your view.

|  | **Strongly Agree** | **Agree** | **Neutral** | **Disagree** | **Strongly Disagree** |
| --- | --- | --- | --- | --- | --- |
| May learn more about health problems that affect them | ⭘ | ⭘ | ⭘ | ⭘ | ⭘ |
| May access potentially more effective treatments for their illness | ⭘ | ⭘ | ⭘ | ⭘ | ⭘ |
| Should get free treatment for their illness^1^ | ⭘ | ⭘ | ⭘ | ⭘ | ⭘ |
| Should receive additional monetary benefits for participating in the research study^2^ | ⭘ | ⭘ | ⭘ | ⭘ | ⭘ |
| May be treated like experimental subjects, not human beings | ⭘ | ⭘ | ⭘ | ⭘ | ⭘ |
| May face some inconveniences (time requirement, blood sampling, treatment side effects, etc.) | ⭘ | ⭘ | ⭘ | ⭘ | ⭘ |
| May be at risk of potentially serious or unexpected side effects | ⭘ | ⭘ | ⭘ | ⭘ | ⭘ |
| May be at risk of their private information being disclosed publicly | ⭘ | ⭘ | ⭘ | ⭘ | ⭘ |

*^1^ Free treatment includes payment for things like lab tests or clinical assessments, drugs or other treatments, travel expenses for any follow up visits and so on – i.e. costs are covered for everything the person would usually have to pay for themselves*

*^2^ This means the person is paid extra money, more than the total costs that they incur, so that they end up making some money out of their participation*

1. What are the potential benefits and risks or burdens of clinical research involving human participants, considered from the perspective of the community? The section refers to broader effects on the community/society. For each question, please tick the box to choose the option that best represents your view.

|  | **Strongly Agree** | **Agree** | **Neutral** | **Disagree** | **Strongly Disagree** |
| --- | --- | --- | --- | --- | --- |
| May make an important contribution to science | ⭘ | ⭘ | ⭘ | ⭘ | ⭘ |
| May provide data or information that improves the health of others in society in some way | ⭘ | ⭘ | ⭘ | ⭘ | ⭘ |
| May stimulate controversial or negative views to develop in the community | ⭘ | ⭘ | ⭘ | ⭘ | ⭘ |
| May have a negative impact on the environment or on the ecological balance in the community | ⭘ | ⭘ | ⭘ | ⭘ | ⭘ |
| Sometimes cost so much to perform that it is not appropriate for LMICs to undertake them on their own^3^ | ⭘ | ⭘ | ⭘ | ⭘ | ⭘ |

*^3^ For example, because funds are diverted from more urgent healthcare needs*

1. Please specify any other benefits or burdens of clinical research involving human subjects that you think are important

|  |
| --- |

1. Thinking about yourself at the present time, what factors might influence your decision on whether or not to participate in a clinical research study? For each question, please tick the box with the option that best represents your view.

|  | **Items** | **Strongly Agree** | **Agree** | **Neutral** | **Disagree** | **Strongly Disagree** |
| --- | --- | --- | --- | --- | --- | --- |
| A | If the research focused on health problems that affect me or my family | ⭘ | ⭘ | ⭘ | ⭘ | ⭘ |
| B | If the research could help a large number of people in general | ⭘ | ⭘ | ⭘ | ⭘ | ⭘ |
| C | If the research question was important from a scientific perspective | ⭘ | ⭘ | ⭘ | ⭘ | ⭘ |
| D | Depending on how safe the study seemed to be | ⭘ | ⭘ | ⭘ | ⭘ | ⭘ |
| E | Depending on how much time I would need to commit to the research | ⭘ | ⭘ | ⭘ | ⭘ | ⭘ |
| F | If all my costs would be covered^1^ | ⭘ | ⭘ | ⭘ | ⭘ | ⭘ |
| G | Depending on how much additional monetary benefit I would get^2^ | ⭘ | ⭘ | ⭘ | ⭘ | ⭘ |
| H | If the ethics of the study had been independently reviewed and approved | ⭘ | ⭘ | ⭘ | ⭘ | ⭘ |
| I | Depending on the reputation of the research team and/or the research institutes involved | ⭘ | ⭘ | ⭘ | ⭘ | ⭘ |
| J | Depending on advice and/or opinions from other people | ⭘ | ⭘ | ⭘ | ⭘ | ⭘ |

*^1^ Includes cover for things like lab tests or clinical assessments, drugs or other treatments, travel expenses for any follow up visits and so on – i.e. costs are covered for everything you would usually have to pay for yourself*

*^2^ Additional monetary benefit means extra money, more than the total costs that you might incur, that is offered to you*

1. Please indicate which three of the above factors would be most important for you when making your decision. Please select the appropriate letter from the above items for your First Priority:……………………………………………………
2. Please indicate which three of the above factors would be most important for you when making your decision. Please select the appropriate letter from the above items for your Second Priority: ………………………………………..
3. Please indicate which three of the above factors would be most important for you when making your decision. Please select the appropriate letter from the above items for your Third Priority: ……….

**SECTION 2**

This section focuses on your perceptions specifically towards **Observational Research** with human involvement. In an observational study, investigators assess health outcomes in groups of participants according to a defined protocol or research plan. As an example, study staff may observe and record the illness course and outcomes in a group of patients in order to learn more about a particular disease. However, these observational activities have no effect on the attending physicians’ decisions on management of the illness (specifically on any drugs or other treatments given). Similarly, individuals’ behavior can be observed or documented and certain outcomes recorded in order to learn about environmental factors that promote health or increase the risk for developing disease.

1. Please tick the box to choose the option that best represents your knowledge/level of understanding about observational research

|  | Excellent –  I have a comprehensive understanding | Good –  I know quite a lot | Average –  I have reasonable knowledge | Poor –  I have very little knowledge | Very Poor –  I do not know anything |
| --- | --- | --- | --- | --- | --- |
| Your knowledge/level of understanding about **Observational Research** | ⭘ | ⭘ | ⭘ | ⭘ | ⭘ |

1. Please tick the box to choose the option that best represents your thoughts about observational research. ***When thinking about observational clinical research, I think ….***

|  | **Strongly Agree** | **Agree** | **Neutral** | **Disagree** | **Strongly Disagree** |
| --- | --- | --- | --- | --- | --- |
| It is important for society that this type of research is carried out | ⭘ | ⭘ | ⭘ | ⭘ | ⭘ |
| It is safe to participate in this type of research | ⭘ | ⭘ | ⭘ | ⭘ | ⭘ |
| I would be happy to participate in this type of research myself | ⭘ | ⭘ | ⭘ | ⭘ | ⭘ |
| For myself, currently, it would be important to get permission from my parents in order for me to participate in this type of research | ⭘ | ⭘ | ⭘ | ⭘ | ⭘ |
| Researchers must check that participants really understand the details of the research (eg, with a short test) before they enroll them. | ⭘ | ⭘ | ⭘ | ⭘ | ⭘ |
| Financial benefits should cover all costs^1^, but no added incentives^2^ should be given | ⭘ | ⭘ | ⭘ | ⭘ | ⭘ |
| Additional incentives^2^ should be given | ⭘ | ⭘ | ⭘ | ⭘ | ⭘ |

*^1^ Including cover for things like lab tests or clinical assessments, drugs or other treatments, travel expenses for any follow up visits and so on – i.e. costs are covered for everything the person would usually have to pay for themselves*

*^2^ This means extra money, more than the total costs that participants incur, so that they end up making some money out of their participation*

1. ***For healthy people***, do you think these groups could be recruited into an **Observational Research study**?

|  | **Strongly Agree** | **Agree** | **Neutral** | **Disagree** | **Strongly Disagree** |
| --- | --- | --- | --- | --- | --- |
| - Babies (<1 yr) | 🌕 | 🌕 | 🌕 | 🌕 | 🌕 |
| - Young children (1-6 yrs) | 🌕 | 🌕 | 🌕 | 🌕 | 🌕 |
| - Older children | 🌕 | 🌕 | 🌕 | 🌕 | 🌕 |
| - Adults (≥18 yrs) | 🌕 | 🌕 | 🌕 | 🌕 | 🌕 |
| - The elderly | 🌕 | 🌕 | 🌕 | 🌕 | 🌕 |

1. ***For people with the disease of interest for the study***, do you think these groups could be recruited into an **Observational Research study**?

|  | **Strongly Agree** | **Agree** | **Neutral** | **Disagree** | **Strongly Disagree** |
| --- | --- | --- | --- | --- | --- |
| - Babies (<1 yr) | 🌕 | 🌕 | 🌕 | 🌕 | 🌕 |
| - Young children (1-6 yrs) | 🌕 | 🌕 | 🌕 | 🌕 | 🌕 |
| - Older children | 🌕 | 🌕 | 🌕 | 🌕 | 🌕 |
| - Adults (≥18 yrs) | 🌕 | 🌕 | 🌕 | 🌕 | 🌕 |
| - The elderly | 🌕 | 🌕 | 🌕 | 🌕 | 🌕 |

1. In addition, do you think the following groups could be recruited into an **Observational Research study**?

|  | **Strongly Agree** | **Agree** | **Neutral** | **Disagree** | **Strongly Disagree** |
| --- | --- | --- | --- | --- | --- |
| Critically ill patients in intensive care units | 🌕 | 🌕 | 🌕 | 🌕 | 🌕 |
| Pregnant women | 🌕 | 🌕 | 🌕 | 🌕 | 🌕 |
| Members of the armed forces | 🌕 | 🌕 | 🌕 | 🌕 | 🌕 |
| Prisoners | 🌕 | 🌕 | 🌕 | 🌕 | 🌕 |
| Individuals with serious mental health issues | 🌕 | 🌕 | 🌕 | 🌕 | 🌕 |

1. Others, please specify and rank: ………………………………………………………………
2. Do you think there are important risks/burdens to **participants in Observational Research studies**. Tick any that you think are relevant:

🞏 Possibility of physical side effects from the research

🞏 Possibility of adverse effects on their mental health from the research

🞏 Possibility of their confidential information being disclosed publicly

🞏 Possibility of adversely affecting their social life (eg. reducing social interactions, affecting relationships)

🞏 Possibility of adversely affecting their economic status

🞏 Possibility of adversely affecting their occupation/studies/schooling

🞏 There are no important risks or burdens associated with observational research

🞏 Other, specify: …………………………………………………………..

1. Do you think there are important risks/burdens to **the community or the environment from Observational Research studies**

⭘ Yes ⭘ No

If yes, please explain what you think these might be: ……………………………………

1. Overall, would you say your views about **Observational Research** are:

|  | **Very positive** | **Somewhat positive** | **Neutral** | **Somewhat negative** | **Very negative** |
| --- | --- | --- | --- | --- | --- |
| Your views | 🌕 | 🌕 | 🌕 | 🌕 | 🌕 |

Please expand on your reasons here: ………………………………………………………

**SECTION 3**

This section focuses on your perception specifically towards **Clinical Trials**, which are research studies done to determine the safety and efficacy of drugs, vaccines, biological products (blood, cells, antibodies, etc.), medical devices, diagnostic products, behavioral treatments and preventive care regimens.

Volunteers taking part in clinical trials may be either patients with the disease of interest or healthy people. They are prospectively assigned to one or more interventions, including the active treatment or a placebo/standard care group to evaluate the effects of the treatment or intervention on human health or behavioral outcomes. The study participants, and sometimes also the study staff, do not know which arm of the trial a particular individual is in, until after the trial concludes and the data have been analyzed.

1. Please tick the box to choose the option that best represents your knowledge/level of understanding about **Clinical Trials**

|  | Excellent –  I have a comprehensive understanding | Good –  I know quite a lot | Average –  I have reasonable knowledge | Poor –  I have very little knowledge | Very Poor –  I do not know anything |
| --- | --- | --- | --- | --- | --- |
| Your knowledge/level of understanding about **Clinical Trials** | ⭘ | ⭘ | ⭘ | ⭘ | ⭘ |

1. Please tick the box to choose the option that best represents your thoughts about **Clinical Trials.** When thinking about **clinical trials**, I think …

|  | **Strongly Agree** | **Agree** | **Neutral** | **Disagree** | **Strongly Disagree** |
| --- | --- | --- | --- | --- | --- |
| It is important for society that this type of research is carried out | ⭘ | ⭘ | ⭘ | ⭘ | ⭘ |
| It is safe to participate in this type of research | ⭘ | ⭘ | ⭘ | ⭘ | ⭘ |
| I would be happy to participate in this type of research myself | ⭘ | ⭘ | ⭘ | ⭘ | ⭘ |
| For myself, currently, it would be important to get permission from my parents in order for me to participate in this type of research | ⭘ | ⭘ | ⭘ | ⭘ | ⭘ |
| Researchers must check that participants really understand the details of the research (eg, with a short test) before they enroll them. | ⭘ | ⭘ | ⭘ | ⭘ | ⭘ |
| Financial benefits should cover all costs^1^, but no added incentives^2^ should be given | ⭘ | ⭘ | ⭘ | ⭘ | ⭘ |
| Additional incentives^2^ should be given | ⭘ | ⭘ | ⭘ | ⭘ | ⭘ |

*^1^ Including cover for things like lab tests or clinical assessments, drugs or other treatments, travel expenses for any follow up visits and so on – i.e. costs are covered for everything the person would usually have to pay for themselves*

*^2^ This means extra money, more than the total costs that participants incur, so that they end up making some money out of their participation*

1. ***For healthy people***, do you think these groups could be recruited into a **Clinical Trial?**

|  | **Strongly Agree** | **Agree** | **Neutral** | **Disagree** | **Strongly Disagree** |
| --- | --- | --- | --- | --- | --- |
| - Babies (<1 yr) | 🌕 | 🌕 | 🌕 | 🌕 | 🌕 |
| - Young children (1-6 yrs) | 🌕 | 🌕 | 🌕 | 🌕 | 🌕 |
| - Older children | 🌕 | 🌕 | 🌕 | 🌕 | 🌕 |
| - Adults (≥18 yrs) | 🌕 | 🌕 | 🌕 | 🌕 | 🌕 |
| - The elderly | 🌕 | 🌕 | 🌕 | 🌕 | 🌕 |

1. ***For people with the disease of interest for the study***, do you think these groups could be recruited into a **Clinical Trial?**

|  | **Strongly Agree** | **Agree** | **Neutral** | **Disagree** | **Strongly Disagree** |
| --- | --- | --- | --- | --- | --- |
| - Babies (<1 yr) | 🌕 | 🌕 | 🌕 | 🌕 | 🌕 |
| - Young children (1-6 yrs) | 🌕 | 🌕 | 🌕 | 🌕 | 🌕 |
| - Older children | 🌕 | 🌕 | 🌕 | 🌕 | 🌕 |
| - Adults (≥18 yrs) | 🌕 | 🌕 | 🌕 | 🌕 | 🌕 |
| - The elderly | 🌕 | 🌕 | 🌕 | 🌕 | 🌕 |

1. In addition, do you think the following groups could be recruited into a **Clinical Trial?**

|  | **Strongly Agree** | **Agree** | **Neutral** | **Disagree** | **Strongly Disagree** |
| --- | --- | --- | --- | --- | --- |
| Critical ill patients in intensive care units | 🌕 | 🌕 | 🌕 | 🌕 | 🌕 |
| Pregnant women | 🌕 | 🌕 | 🌕 | 🌕 | 🌕 |
| Members of the armed forces | 🌕 | 🌕 | 🌕 | 🌕 | 🌕 |
| Prisoners | 🌕 | 🌕 | 🌕 | 🌕 | 🌕 |
| Individuals with serious mental health issues | 🌕 | 🌕 | 🌕 | 🌕 | 🌕 |

1. Others, please specify and rank: ………………….
2. Do you think there are other important risks/burdens to **participants in Clinical Trials**. Tick any that you think are relevant:

🞏 Possibility of physical side effects from the research

🞏 Possibility of adverse effects on their mental health from the research

🞏 Possibility of their confidential information being disclosed publicly

🞏 Possibility of adversely affecting their social life (eg. reducing social interactions, affecting relationships)

🞏 Possibility of adversely affecting their economic status

🞏 Possibility of adversely affecting their occupation/studies/schooling

🞏 There are no important risks or burdens associated with clinical trials

🞏 Other, specify: …………………………………………………………..

1. Do you think there are important risks/burdens to **the community or the environment from Clinical Trials**

⭘ Yes ⭘ No

If yes, please explain what you think these might be: ……………………………………

1. Overall, would you say your views about **Clinical Trials** are:

|  | **Very positive** | **Somewhat positive** | **Neutral** | **Somewhat negative** | **Very negative** |
| --- | --- | --- | --- | --- | --- |
| Your views | 🌕 | 🌕 | 🌕 | 🌕 | 🌕 |

Please expand on your reasons here: …………………………………………………………

**SECTION 4**

This section focuses on your perceptions specifically towards **Human Challenge Trials**.

**Human Challenge Trials**: A human challenge trial is a very particular type of clinical trial, which involves healthy volunteers only. The volunteers are deliberately infected with a disease pathogen, and then their responses are observed in great detail. If the study participant becomes ill they receive the best available treatment for their illness. The challenge organism may be similar to the wild-type organism and therefore potentially quite pathogenic, or may have been adapted/attenuated from wild-type to reduce pathogenicity, or may have been genetically modified in some manner. These trials can provide a powerful scientific method for testing of vaccines and therapeutics and for studying human-pathogen interactions in small numbers of healthy volunteers.

1. Please tick the box to choose the option that best represents your knowledge/level of understanding about **Human Challenge Trials**

|  | Excellent –  I have a comprehensive understanding | Good –  I know quite a lot | Average –  I have reasonable knowledge | Poor –  I have very little knowledge | Very Poor –  I do not know anything |
| --- | --- | --- | --- | --- | --- |
| Your knowledge/level of understanding about **Human Challenge Trials** | ⭘ | ⭘ | ⭘ | ⭘ | ⭘ |

*

1. Please tick the box to choose the option that best represents your thoughts about **Human Challenge Trials**. When thinking about **Human Challenge Trials**, I think ….

|  | **Strongly Agree** | **Agree** | **Neutral** | **Disagree** | **Strongly Disagree** |
| --- | --- | --- | --- | --- | --- |
| It is important for society that this type of research is carried out | ⭘ | ⭘ | ⭘ | ⭘ | ⭘ |
| It is safe to participate in this type of research | ⭘ | ⭘ | ⭘ | ⭘ | ⭘ |
| I would be happy to participate in this type of research myself | ⭘ | ⭘ | ⭘ | ⭘ | ⭘ |
| For myself, currently, it would be important to get permission from my parents in order for me to participate in this type of research | ⭘ | ⭘ | ⭘ | ⭘ | ⭘ |
| Researchers must check that participants really understand the details of the research (eg, with a short test) before they enroll them. | ⭘ | ⭘ | ⭘ | ⭘ | ⭘ |
| Financial benefits should cover all costs^1^, but no added incentives^2^ should be given | ⭘ | ⭘ | ⭘ | ⭘ | ⭘ |
| Additional incentives^2^ should be given | ⭘ | ⭘ | ⭘ | ⭘ | ⭘ |

*^1^ Including cover for things like lab tests or clinical assessments, drugs or other treatments, travel expenses for any follow up visits and so on – i.e. costs are covered for everything the person would usually have to pay for themselves*

*^2^ This means extra money, more than the total costs that participants incur, so that they end up making some money out of their participation*

1. ***For healthy people***, do you think these groups could be recruited into a **Human Challenge Trial**?

|  | **Strongly Agree** | **Agree** | **Neutral** | **Disagree** | **Strongly Disagree** |
| --- | --- | --- | --- | --- | --- |
| - Babies (<1 yr) | 🌕 | 🌕 | 🌕 | 🌕 | 🌕 |
| - Young children (1-6 yrs) | 🌕 | 🌕 | 🌕 | 🌕 | 🌕 |
| - Older children | 🌕 | 🌕 | 🌕 | 🌕 | 🌕 |
| - Adults (≥18 yrs) | 🌕 | 🌕 | 🌕 | 🌕 | 🌕 |
| - The elderly | 🌕 | 🌕 | 🌕 | 🌕 | 🌕 |

1. In addition, do you think these groups could be recruited into a **Human Challenge Trial**?

|  | **Strongly Agree** | **Agree** | **Neutral** | **Disagree** | **Strongly Disagree** |
| --- | --- | --- | --- | --- | --- |
| Pregnant women | 🌕 | 🌕 | 🌕 | 🌕 | 🌕 |
| Members of the armed forces | 🌕 | 🌕 | 🌕 | 🌕 | 🌕 |
| Prisoners | 🌕 | 🌕 | 🌕 | 🌕 | 🌕 |
| Individuals with serious mental health issues | 🌕 | 🌕 | 🌕 | 🌕 | 🌕 |

1. Others, please specify and rank: ……………………………………………………………
2. Do you think there are other important risks/burdens to **participants in Human Challenge Trials**. Tick any that you think are relevant:

🞏 Possibility of physical side effects from the research

🞏 Possibility of adverse effects on their mental health from the research

🞏 Possibility of their confidential information being disclosed publicly

🞏 Possibility of adversely affecting their social life (eg. reducing social interactions, affecting relationships)

🞏 Possibility of adversely affecting their economic status

🞏 Possibility of adversely affecting their occupation/studies/schooling

🞏 There are no important risks or burdens associated with human challenge trials

🞏 Other, specify: …………………………………………………………..

1. Do you think there are important risks/burdens to **the community or the environment from Human Challenge Trials**

⭘ Yes ⭘ No

If yes, please explain what you think these might be: ……………………………………

1. Overall, would you say your views about **Human Challenge Trials** are:

|  | **Very positive** | **Somewhat positive** | **Neutral** | **Somewhat negative** | **Very negative** |
| --- | --- | --- | --- | --- | --- |
| Your views | 🌕 | 🌕 | 🌕 | 🌕 | 🌕 |

Please expand on your reasons here: …………………………………………………………..

***Thank you very much for your contribution by completion of this questionnaire***
