# Supplementary material for: Vietnamese University Students’ Perceptions and Attitudes Toward Participation in Clinical Research: Mixed Methods Study
Source: J Particip Med. 2026 Feb 12;18:e86269. doi: 10.2196/86269 (PMC12946779; doi:10.2196/86269)
Supplement: Multimedia Appendix 2 [file jopm_v18i1e86269_app2.docx]

**
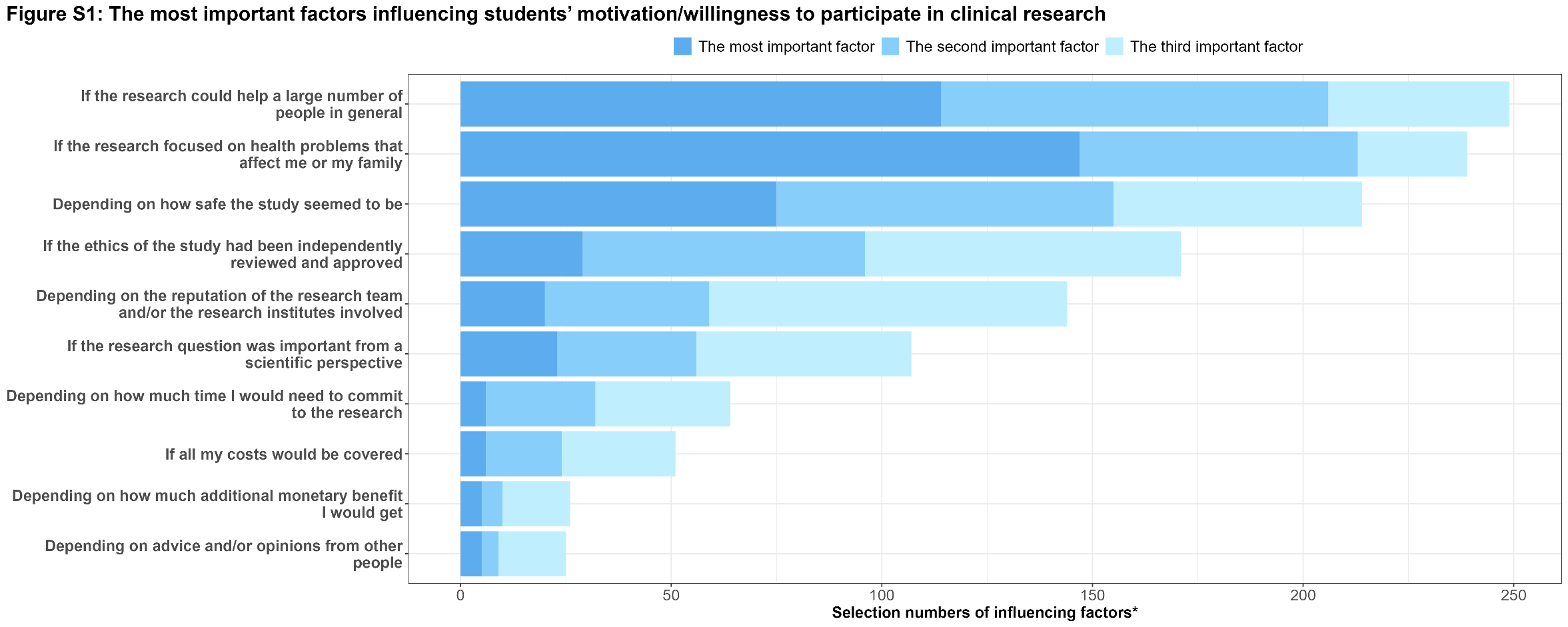
**

**Note: We asked students to select the top three factors that they would consider when deciding whether to participate in a clinical research study. The horizontal bars represent the total number of times each factor was selected, ranked in order from most to least frequent.*

**Table S1.** **Demographic characteristics of SEED cohort participants included in this analysis compared to those of students initially invited to join the cohort.**

|  | **Students invited to join the SEED cohort**  **N=1203** | | **SEED cohort students included in this analysis**  **n=437** | |
| --- | --- | --- | --- | --- |
| **Age in years^1^ (mean)** | 19 (SD=±1) | | 19 (SD=±1) | |
| Range | 18 – 30 | | 18 – 25 | |
|  |  |  |  |  |
| **Faculty^1,2^** |  |  |  |  |
| General medicine | 907 | (76) | 302 | (69) |
| Public health | 288 | (24) | 135 | (31) |
|  |  |  |  |  |
| **Gender^1^** |  |  |  |  |
| Female | 558 | (46) | 216 | (49) |
| Male | 643 | (54) | 221 | (51) |
|  |  |  |  |  |
| **Academic year at enrollment ^1^** |  |  |  |  |
| First year | 696 | (58) | 254 | (58) |
| Third year | 498 | (42) | 183 | (42) |
| Others | 5 | (0) | 0 | (0) |
|  |  |  |  |  |
|  |  |  |  |  |

***^1^*** *Missing data: for age=6; for faculty=8; department=8; for gender=2; for academic year at enrolment=4.*

*^2^ The Chi-square test result for the proportion of students in different faculties was significant (p-value < 0.001).*

## **Table S2. Characteristics of students participating in IDIs and FGDs.**

|  | | **Students participating in FGDs and IDIs** | | **SEED cohort students included in this study** | |
| --- | --- | --- | --- | --- | --- |
| **Number of students** | | **n=74** | | **N=437** | |
| **Age in years (mean)** | | 20 (SD = ± 2) | | 19 (SD=±1) | |
| Range | | 18 - 25 | | 18 - 25 | |
| **Gender** | Male | 39 | (53) | 221 | (51) |
| **Faculty** | General medicine | 63 | (85) | 302 | (69) |
|  | Public health | 11 | (15) | 135 | (31) |
| **Academic year at enrolment** | First year | 31 | (42) | 254 | (58) |
|  | Third year | 43 | (58) | 183 | (42) |
| **Ethnicity** | Kinh | 65 | (88) | 392 | (90) |
|  | Hoa | 3 | (4) | 22 | (5) |
|  | Others | 6 | (8) | 21 | (5) |
| **Family monthly income^2^** | Less than 3 million VND | 2 | (3) | 8 | (2) |
|  | 3-10 million VND | 18 | (24) | 100 | (24) |
|  | 10-60 million VND | 26 | (35) | 155 | (37) |
|  | Over 60 million VND | 1 | (1) | 9 | (2) |
|  | Do not know | 16 | (22) | 92 | (22) |
|  | Prefer not to answer | 11 | (15) | 59 | (14) |
| **Socio-economic status^3^** | Poor | 7 | (9) | 27 | (6) |
|  | Average | 60 | (81) | 346 | (81) |
|  | Wealthy | 0 | (0) | 2 | (0) |
|  | Prefer not to answer | 7 | (9) | 52 | (12) |

^2^: VND refers to Vietnamese Dong. 1 VND = 0.00004328 USD (Average VND to USD exchange rate in December 2020). For the family income bands, we applied the Vietnamese government's poverty threshold for 2016 – 2020 (Vietnam Government, 2015) and the 21-times disparity in income between the poorest and richest populations identified by Oxfam in their 2017 survey [29]. We assumed that most households had two earners. The family monthly income below 3 million VND was considered poor, and above 60 million VND was considered wealthy. Between these two thresholds we divided household income bands into two categories: income between 3 and 10 million VND was considered as low normal, and between 10-60 million VND that was grouped as high normal.

^3^: Socio-economic status refers to the students’ personal assessment of their family’s overall socio-economic status within the Vietnamese context. **Table S3. Factors influencing students’ decision to participate in clinical research by faculty and academic year at enrolment (n=437).**

|  | **Faculty** | | | | | | **p - value^a^** | **Adjusted p – value^b^** | **Academic year at enrolment** | | | | | | | **p -value^a^** | **Adjusted p – value^b^** |
| --- | --- | --- | --- | --- | --- | --- | --- | --- | --- | --- | --- | --- | --- | --- | --- | --- | --- |
|  | **General medicine**  **n=302** | | | **Public health**  **n=135** | | |  |  | **First-year students**  **n=254** | | | | **Third-year students**  **n=183** | | |  |  |
|  | ***d*** | ***n*** | ***a*** | ***d*** | ***n*** | ***a*** |  |  | ***d*** | ***n*** | ***a*** | ***d*** | | ***n*** | ***a*** |  |  |
| If the research focused on health problems that affect me or my family | 17  (6) | 44 (15) | 241 (80) | 7  (5) | 33 (24) | 95 (70) | **0.043*** | 0.280 | 16 (6) | 51 (20) | 187 (74) | 8  (4) | | 26 (14) | 149 (81) | 0.162 | 0.272 |
| If the research could help a large number of people in general | 3  (1) | 22 (7) | 277 (92) | 2  (1) | 18 (13) | 115 (85) | 0.112 | 0.280 | 4  (2) | 25 (10) | 225 (88) | 1  (1) | | 15 (8) | 167 (91) | 0.501 | 0.557 |
| If the research question was important from a scientific perspective^1^ | 7  (2) | 51 (17) | 243 (81) | 4  (3) | 21 (16) | 109 (81) | 0.880 | 0.894 | 5  (2) | 42 (17) | 205 (81) | 6  (3) | | 30 (17) | 147 (80) | 0.697 | 0.697 |
| Depending on how safe the study seemed to be^1^ | 3  (1) | 35 (12) | 262 (87) | 1  (1) | 27 (20) | 107 (79) | 0.069 | 0.280 | 4  (2) | 41 (16) | 209 (82) | 0  (0) | | 21 (12) | 160 (88) | 0.089 | 0.223 |
| Depending on how much time I would need to commit to the research | 6  (2) | 52 (17) | 244 (81) | 2  (1) | 33 (25) | 100 (74) | 0.205 | 0.342 | 5  (2) | 64 (25) | 185 (73) | 3  (2) | | 21 (11) | 159 (87) | **0.001*** | **0.010*** |
| If all my costs would be covered | 20  (7) | 101 (33) | 181 (60) | 8  (6) | 48 (35) | 79 (59) | 0.894 | 0.894 | 19 (7) | 97 (38) | 138 (54) | 9  (5) | | 52 (28) | 122 (67) | **0.034*** | 0.170 |
| Depending on how much additional monetary benefit I would get | 39  (13) | 156 (52) | 107 (35) | 8  (6) | 78 (58) | 49 (36) | 0.086 | 0.280 | 32 (12) | 139 (55) | 83 (33) | 15  (8) | | 95 (52) | 73 (40) | 0.163 | 0.272 |
| If the ethics of the study had been independently reviewed and approved | 3  (1) | 34 (11) | 265 (88) | 1  (1) | 19 (14) | 115 (85) | 0.689 | 0.861 | 3  (1) | 38 (15) | 213 (84) | 1  (1) | | 15 (8) | 167 (91) | 0.076 | 0.223 |
| Depending on the reputation of the research team and/or the research institutes involved | 2  (0) | 26 (9) | 274 (91) | 1  (1) | 20 (15) | 114 (84) | 0.147 | 0.294 | 2  (1) | 32 (12) | 220 (87) | 1  (1) | | 14 (8) | 168 (92) | 0.236 | 0.337 |
| Depending on advice and/or opinions from other people | 25  (8) | 99 (33) | 178 (50) | 9  (7) | 52 (38) | 74 (55) | 0.478 | 0.683 | 23 (9) | 84 (33) | 147 (58) | 11  (6) | | 67 (37) | 105 (57) | 0.437 | 0.546 |

*Note: d=disagree, n=neutral, a=agree;*

*^1^ Missing data=2*

*^a^ p-values were calculated using the Chi-square test; * Significant outcomes (p-value <0.05) are printed in bold.*

*^b^ Adjusted p-values were calculated using the* *Benjamini and Hochberg correction method for multiple testing.*
